# Proteomics profiling of urine with surface enhanced laser desorption/ionization time of flight mass spectrometry

**DOI:** 10.1186/1477-5956-5-2

**Published:** 2007-01-15

**Authors:** Han Roelofsen, Gloria Alvarez-Llamas, Marianne Schepers, Karloes Landman, Roel J Vonk

**Affiliations:** 1 University of Groningen, University Medical Centre Groningen, Centre for Medical Biomics, Groningen, The Netherlands

## Abstract

**Background:**

Urine consists of a complex mixture of peptides and proteins and therefore is an interesting source of biomarkers. Because of its high throughput capacity SELDI-TOF-MS is a proteomics technology frequently used in biomarker studies. We compared the performance of seven SELDI protein chip types for profiling of urine using standard chip protocols.

**Results:**

Performance was assessed by determining the number of detectable peaks and spot to spot variation for the seven array types and two different matrices: SPA and CHCA. A urine sample taken from one healthy volunteer was applied in eight-fold for each chip type/matrix combination. Data were analyzed for total number of detected peaks (S/N > 5). Spot to spot variation was determined by calculating the average CV of peak intensities. In addition, an inventory was made of detectable peaks with each chip and matrix type. Also the redundancy in peaks detected with the different chip/matrix combinations was determined. A total of 425 peaks (136 non-redundant peaks) could be detected when combining the data from the seven chip types and the two matrices. Most peaks were detected with the CM10 chip with CHCA (57 peaks). The Q10 with CHCA (51 peaks), SEND (48 peaks) and CM10 with SPA (48 peaks) also performed well. The CM10 chip with CHCA also has the best reproducibility with an average CV for peak intensity of 13%.

**Conclusion:**

The combination of SEND, CM10 with CHCA, CM10 with SPA, IMAC-Cu with SPA and H50 with CHCA provides the optimal information from the urine sample with good reproducibility. With this combination a total of 217 peaks (71 non-redundant peaks) can be detected with CV's ranging from 13 to 26%, depending on the chip and matrix type. Overall, CM10 with CHCA is the best performing chip type.

## Background

Urine is an easily accessible body fluid which makes it an interesting source of biomarkers for clinical and population studies. Urine contains a complex mixture of proteins and peptides and can be seen as the reflection of serum composition and kidney function [1]. Protein content of urine is relatively low compared to serum. Norden et al. [2] estimated the excretion of proteins > 10 kDa on 33.7 ± 10.7 mg/24 hrs. Peptide excretion in the range of 750 Da to 10 kDa was 22.0 ± 9.6 mg/24 hrs. Protein composition of urine appears to be less complex than serum. Spahr et al. [3] and Davis et al. [4] could identify 124 proteins by LC-MS/MS of a tryptic digest of urine. Oh et al. [5] could annotate 113 different urinary proteins by 2D gel in combination with depletion of high abundant proteins. Pieper et al. [6] identified 150 unique proteins using a similar approach. There is little overlap in the proteins identified by the different methods employed [7]. Sun et al. [7] could identify a total of 226 urinary proteins by combining results of different fractionation and mass spectrometry approaches. Although the proteomics methods mentioned above have a high resolving power, they generally have a low throughput. For clinical or epidemiological biomarker studies, where hundreds of samples are compared, a high throughput proteomics technology is essential. Because of its high throughput capacity, SELDI-TOF-MS is often used in these types of studies. SELDI uses proteinchip arrays with different affinity surfaces such as hydrophobic chip types (SEND, H50 and H4), weak cation exchange (WCX, CM10), anion exchange (SAX2, Q10), copper-coated IMAC chips and silica coated chips (NP20). These can be used to reduce the sample complexity which facilitates the detection of more proteins [8]. Several papers have been published thus far in which one chip type or a combination of chip types were used for SELDI profiling of urine. For example, Schaub et al. [9] and Nguyen et al. [10] used NP20, Clarke et al used IMAC and H4 chips [11], Zhang et al. used IMAC chips [12], Dare et al. used SAX2 and H4 [13], Neuhoff et al. used WCX chips [14], Traum et al. [15] and Khurana et al. [16] used CM10 and IMAC chips and Voshol et al. used CM10 [17]. Woroniecki et al. [18] used a combination of CM10, Q10 H50 and IMAC-30 chips. In these studies the different chip types were processed either with SPA or CHCA as matrix. In this manuscript we describe a study into the performance of the different chip types for profiling of urine using standard chip protocols. We investigated intra-chip variability, the number of detected peaks and the redundancy in detected peaks for the different array types and two matrices (SPA and CHCA). The results facilitate the set-up of large scale SELDI-based biomarker studies with urine.

## Results and discussion

**Figure 1:**
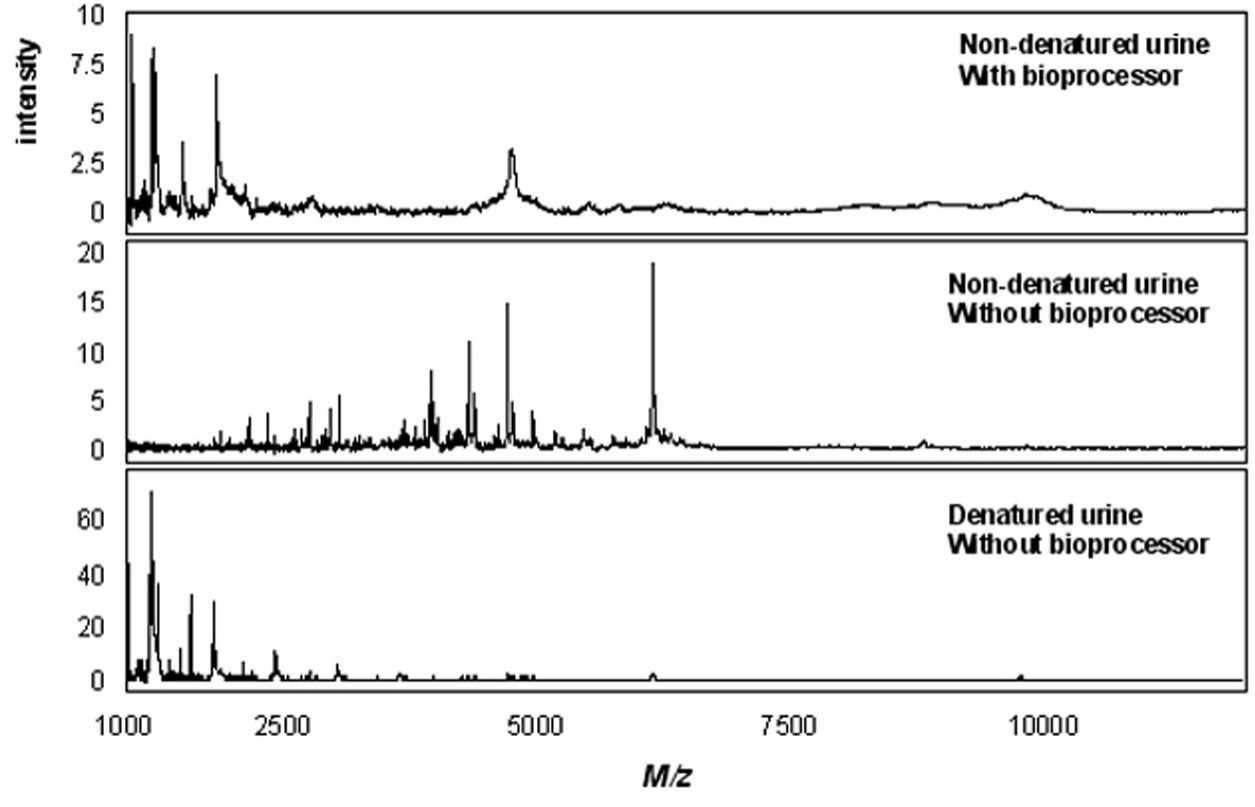
**Effect of the use of a bioprocessor and sample denaturation on the urinary protein profile obtained with a SEND chip**. 200 μl of 2-fold diluted non-denatured urine in 0.1% TFA was incubated using the bioprocessor (upper trace); 2 μl of non-denatured urine was directly applied to the spot without the use of a bioprocessor (middle trace); 2 μl of denatured urine directly applied to the spot without the use of a bioprocessor (lower trace). The same concentration was applied to all three conditions.

For a comparison of SELDI chip performance with urine, standard chip protocols as provided by Ciphergen were applied. In these protocols the use of a bioprocessor is recommended for all chip types except SEND and NP20. The bioprocessor is a 96-well format cassette that can maximally hold 12 chips and enables the incubation and washing of the spots with larger volumes and facilitates the incubation and washing steps. For NP20 and SEND chips no standard bioprocessor protocols are provided. Therefore, we evaluated the performance of these chip types with a bioprocessor set-up. However, as shown in Figure 1, they performed poorly with this set-up. As an example, figure 1 (upper trace) shows a spectrum of non-denatured urine obtained with a SEND chip and bioprocessor. When the same concentration of urine was applied directly on the spot without making use of a bioprocessor, the spectra improved considerable in terms of number of detectable peaks (middle trace). Denaturation of urine had a deteriorating effect on the spectra obtained with the SEND chip (figure 1, lower trace). This was also observed for the H4 chip (not shown). The reason for this is unknown. However, the other chip types performed better with denatured urine. Therefore, only SEND and H4 chips were processed using non-denatured urine. Details of the protocols are provided in the methods section.

**Figure 2:**
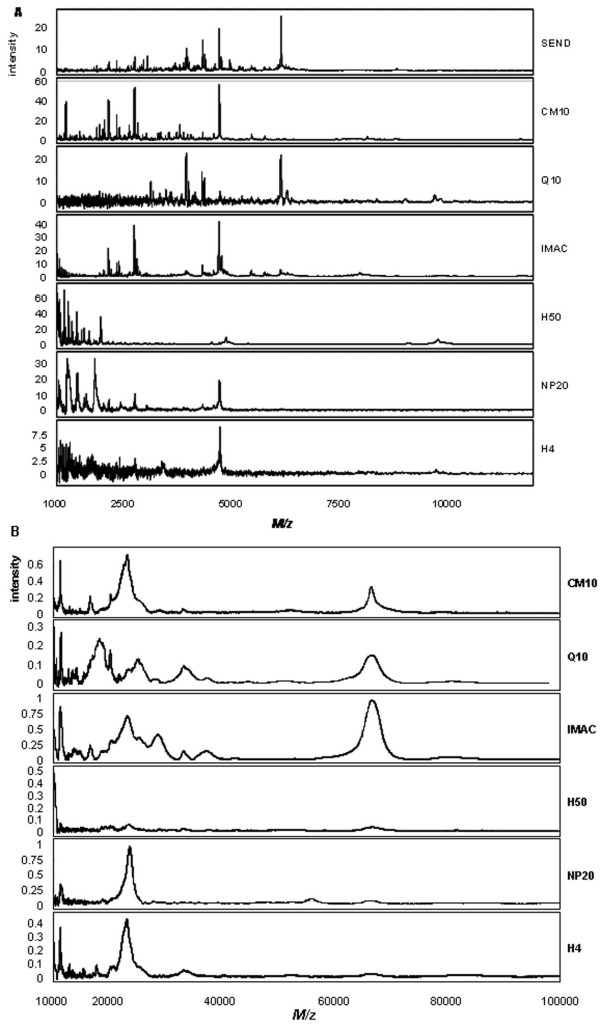
**Urinary protein profiles obtained with the different chip types**. (A) Examples of urinary protein profiles for the 1 – 10 kDa mass range obtained with the different chip types as indicated and CHCA as matrix. (B) Examples of urinary protein profiles for the 10 – 100 kDa mass range obtained with the different chip types as indicated and SPA as matrix.

Protein profiles were generated by SELDI-TOF-MS. To test intra-chip variability in terms of detected peaks the same urine sample was applied to eight spots on one chip for each chip type/matrix combination. SEND and CHCA chips were measured in the mass range of 1 to 30 kDa (no peaks could be detected above 30 kDa with this matrix type) and SPA treated chips were measured in the mass range of 1 to 100 kDa. Figure 2A shows examples of spectra obtained in the 1 to 12 kDa mass range for CHCA. Especially the H4, but also the Q10 spectra with CHCA, suffer from considerable noise in the low mass region. H50 and NP20 mainly show peaks below 3000 Da. Figure 2B depicts examples of spectra obtained with the different chip types for the mass range of 10 to 100 kDa for SPA. The SEND chip contains covalently bound CHCA that reduces the chemical noise in the low mass region and therefore cannot be used in combination with SPA as matrix. The H50 chip performs poorly with SPA.

**Figure 3:**
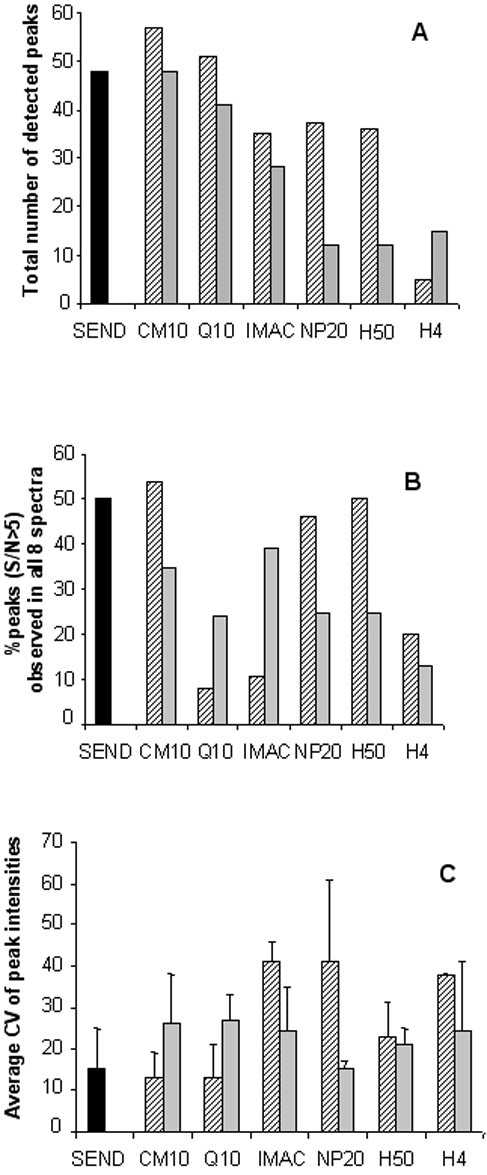
**Assessment of chip performance**. Overview of total peak number (panel A; S/N>5); percentage of peaks observed in all eight spectra with a S/N > 5 as a measure of reliability of the measurement (panel B) and the average CV (peak intensity) of those peaks detected in all eight spectra with a S/N>5 (panel C). Hatched bars represent CHCA, grey bars represent SPA and the black bar represents the SEND chip with covalently coupled CHCA. The different chip types used are indicated. Mass range for CHCA is 1–30 kDa, Mass range for SPA is 1–100 kDa.

From the eight spectra obtained per chip and matrix type, total number of peaks detected with a S/N>5 in one or more of the eight spectra was determined (figure 3A). Best chip types in terms of detectable peaks are CM10 with CHCA (57 peaks), Q10 with CHCA (51 peaks), SEND (48 peaks) and CM10 with SPA (48 peaks). Another aspect of chip performance is the reliability of the measurement. Chip type/matrix combinations that yield spectra containing a relatively high number of peaks with high signal to noise ratios will give more reliable results than combinations that yield spectra with a relatively low number of high signal to noise ratio peaks. To determine this we calculated for each chip type/matrix combination the fraction of the peaks that could be detected on all eight spots with a S/N> 5. As shown in figure 3B, CM10 with CHCA (54% in all eight spectra), SEND (50% in all eight spectra), H50 with CHCA (50% in all eight spectra) and NP20 with CHCA (46% in all eight spectra) score best on this aspect. In contrast, the Q10 chip, which has a relatively high total peak number of 51 and 41 (figure 3A) for CHCA and SPA, respectively, scores poorly on this feature with only 8% and 24% for CHCA and SPA, respectively. This indicates that for urine the Q10 chip has a relative large variation in signal to noise ratios between spots compared to other chip types. The percentages of peaks that have, in all replicates, signal to noise ratios higher than a certain cut-off value (in this case S/N> 5) can therefore be considered as an indicator for chip performance. The percentage value will depend on the chosen cut-off signal to noise value. To determine intra-chip spot to spot variation in peak intensities the average CV was calculated for peaks with a S/N>5 in all eight spectra (figure 3C). The CM10 chip with CHCA and the SEND chip performed the best with average CV's of 13% and 15%, respectively. Although Q10 with CHCA and NP20 with SPA also have low CV's of 13% and 15%, respectively, these values were based on only 4 and 3 peaks, respectively.

**Figure 4:**
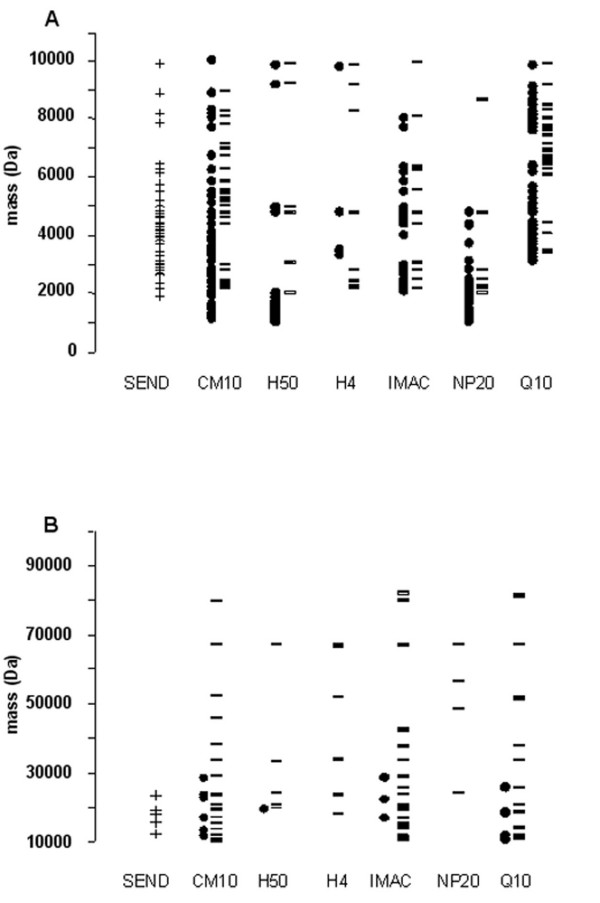
**Distribution of the detected masses for the different chip types**. Distribution of the detected masses over the 1–10 kDa (panel A) and 10–100 kDa mass range (panel B). Black dots represent masses detected with CHCA, black stripes represent masses detected with SPA and the crosses represent masses detected with the SEND chip with covalently coupled CHCA. The different chip types used are indicated.

Besides peak count and reproducibility, also the distribution of the detected masses over the mass range was investigated as a parameter for chip performance. The distribution of the detected peaks (S/N >5) over the 1–100 kDa mass range was plotted for the different chip and matrix types (figure 4). The evaluated mass range (1–100 kDa) was split into two ranges for a better visualization of the masses distribution. For the low mass range (from 1 to 10 kDa), CM10 with CHCA (51 peaks) and SEND (43 peaks) show the most peaks (figure 4A). H50 with CHCA (30 peaks) and NP20 (27 peaks) with CHCA are especially suitable in detecting peaks in the 1 to 2 kDa range. For the high mass range (10 to 100 kDa, figure 4B), CM10 with SPA (20 peaks) and IMAC with SPA (17 peaks) show the most peaks.

With the seven chip types and two matrix types, we were able to detect 425 peaks (S/N > 5) in total. However, there is considerable overlap in detected peaks between the different chip types. In Table 1 we estimated this redundancy using a 0.3% mass variation, which is the standard value used for cluster analyses of SELDI spectra. In total, 136 non-redundant peaks can be detected when combining all chip types measured with CHCA as well as SPA, not taken into account possible doubly charged ions. Most non-redundant peaks were detected with the CM10 chip for CHCA (20 peaks) as well as SPA (17 peaks) and the Q10 chip for CHCA (22 peaks) as well as SPA (14 peaks). The H50 chip with CHCA also detects a considerable number of non-redundant peaks (15 peaks). H4 with CHCA (0 peaks) and H4 with SPA (2 peaks), NP20 with SPA (3 peaks) and H50 with SPA (4 peaks) do not provide much extra information which can not be obtained from other chip and matrix combinations and therefore are of less importance to use for profiling of urine.

**Table 1:** Peak number and redundancy per chip and matrix type.

		**SEND**	**CM10**		**H50**		**H4**		**IMAC30**		**NP20**		**Q10**		
			**CHCA**	**SPA**	**CHCA**	**SPA**	**CHCA**	**SPA**	**CHCA**	**SPA**	**CHCA**	**SPA**	**CHCA**	**SPA**	**Total**
**Nr of peaks**		48	57	48	36	12	5	15	35	28	37	12	51	41	425
**SEND**			15	11	1	0	1	3	14	6	5	2	10	4	
**CM10**	**CHCA**	15		10	6	0	0	2	15	5	10	1	5	4	
	**SPA**	11	10		1	2	1	7	8	14	4	3	1	7	
**H50**	**CHCA**	1	6	1		1	0	2	2	2	14	1	0	0	
	**SPA**	0	0	2	1		1	2	1	2	0	4	1	4	
**H4**	**CHCA**	1	0	1	0	1		1	0	0	0	1	2	2	
	**SPA**	3	2	7	2	2	1		4	3	1	4	2	3	
**IMAC30**	**CHCA**	14	15	8	2	1	0	4		4	6	3	8	2	
	**SPA**	6	5	14	2	2	0	3	4		3	2	2	4	
**NP20**	**CHCA**	5	10	4	14	0	0	1	6	3		2	2	0	
	**SPA**	2	1	3	1	4	1	4	3	2	2		2	1	
**Q10**	**CHCA**	10	5	1	0	1	2	2	8	2	2	2		7	
	**SPA**	4	4	7	0	4	2	3	2	4	0	1	7		
**Non-redundant peaks**		11	20	17	15	4	0	2	9	8	11	3	22	14	136

a) Peak number was determined using a threshold of S/N >5. Redundancy was calculated using a 0.3% mass variation. Mass range for CHCA is 1–30 kDa, Mass range for SPA is 1–100 kDa.

## Conclusion

Based on the results of this study, SEND, CM10 with CHCA, CM10 with SPA, IMAC-Cu with SPA and H50 with CHCA are the best performing combinations of chip and matrix for protein profiling of urine with SELDI. When urinary profiles generated from these chip/matrix types are combined optimal information from a urine sample with good reproducibility is obtained. A total of 217 peaks (71 non-redundant peaks) can be detected with this combination with CV's for peak intensity ranging from 13 to 26%, depending on the chip and matrix type (see figure 3B). This CV range for peak intensity is similar to that previously reported for SELDI analyses of serum and plasma [19,20]. If the use of only one chip type is favored, the CM10 chip is the best choice for urine profiling. Figure 3 indicates that with CHCA as well as with SPA this chip type yields relatively high total peak numbers (57 and 48, respectively) and low CV's for peak intensity (figure 3B). Furthermore, a considerable number of peaks are only detected with this chip type (see table 1). If profiling of urine is intended in a particular mass range, several considerations can be taken into account. The H50 chip with CHCA is especially useful in the low mass range (below 2000 Da) while the IMAC-Cu chip with SPA is especially useful in the 10 to 100 kDa mass range (figure 4). The SEND chip performs best in the 2 to 7 kDa range but it is not performing optimally with a bioprocessor set-up (figure 1). Therefore, this chip type can not be easily incorporated into an automated protocol using a pipetting robot, as is normally the case in medium to large scale biomarker studies. The chip protocols used in this study are general and may need further refinement depending on specific questions or interest in a certain mass range. The results of this study may improve the outcome of SELDI based urinary biomarker studies by facilitating choices for (combinations of) chip and matrix types.

## Materials and methods

### Sample pretreatment

Fresh human morning urine from a healthy male volunteer was centrifuged for 5 minutes at 7000 g and 4°C to remove debris. The supernatant was used for further analyses. Denatured urine was used for all chip types except for SEND and H4. For denaturation, 160 μl of urine was mixed with 60 μl of 9 M urea/2% CHAPS in 50 mM Tris pH 9,0. The mixture was incubated at 4°C for 30 minutes under continuous shaking.

### Chip handling protocols

For the seven chip types tested, chip protocols used were based on standard protocols provided by the chip manufacturer Ciphergen Biosystems. The sample was applied manually to eight spots per condition (seven chip types prepared with two matrix types, except for SEND which has CHCA already incorporated as matrix). All chip types were processed in a bioprocessor, except SEND and NP20. The bioprocessor is a 96-well format cassette that can maximally hold 12 chips and enables the incubation and washing of the spots with larger volumes. The SEND chip was activated by incubating the spots with 5 μl of 0.1% TFA for 30 seconds without agitation. This was repeated once. Then, 10 μl non-denatured urine was mixed with 10 μl 0.2% TFA and 2 μl of sample was applied per spot. The array was incubated for 10 minutes in a humidity chamber (closed box with wet tissue) at room temperature. Thereafter, the samples were removed and spots were washed with 5 μl of 0.1% TFA for 30 seconds. Then, 2 μl of 25% ACN (v/v) and 0.1% TFA in water was added and spots were allowed to air-dry. No matrix addition is necessary since the SEND chip surface contains covalently linked CHCA.

Spots on a NP20 chip were incubated for 20 minutes with 5 μl of denatured urine in a humidity chamber. Thereafter, spots were washed three times with 5 μl of ultra pure water and were air-dried. Then, two times 0.5 μl of matrix solution was applied. A saturated solution of SPA and a 5-fold diluted saturated solution of CHCA in 50% ACN (v/v), 0.5% TFA (v/v) were used.

For the other chip types a bioprocessor was used for incubation and washing steps. As a first step before addition of binding buffer, some chip types (H50, H4 and IMAC30) require activation as detailed below. The spots of the H50 chip were activated by washing twice with 5 μl 50 % ACN/water for 5 minutes. The chip was then air-dried for 15 minutes. Spots on the H4 array were pre-treated with 5 μl ACN and air-dried. The spots on the IMAC30 chip were loaded with 50 μl of 100 mM CuSO_4 _and incubated for 5 minutes. Thereafter, they were washed twice for 5 minutes with 200 μl ultra pure water, once with 50 μl of 100 mM NH_4_Ac pH 4.0 and once with 200 μl water. Next, for all chip types, 200 μl binding buffer was added to the wells. The buffer composition depends on the chip type. This was: for CM10, 100 mM NH_4_Ac pH 4.0 + 0.05% Triton; for Q10, 100 mM Tris HCl pH 10 + 0.05 % Triton; for IMAC, 0.5 M NaCl in PBS pH 7.4 + 0.1 % Triton; for H50, 10 % ACN, 0.1% TFA in PBS pH 7.4; for H4 10 % ACN, 0.25 M NaCl in PBS pH 7.4. The spots were incubated for 5 minutes at room temperature with agitation (250 rpm). This was repeated once. The buffer was removed and replaced by 160 μl binding buffer and 40 μl of urine (denatured, except for H4) and incubated for 30 minutes. After incubation, the sample was removed and spots were washed three times for 5 minutes with 200 μl binding buffer and once with 200 μl ultra pure water. After removal of water, the chip was allowed to dry before applying two times 0.5 μl of matrix solution, prepared as described above.

### Data acquisition and processing

Protein profiles were generated in a SELDI-TOF-MS (Ciphergen Biosystems). SEND and CHCA chips were measured in the mass range of 1 to 30 kDa. SPA treated chips were measured in the mass range of 1 to 100 kDa. 91 transients were averaged in the final spectra. Source voltage was 20000 V, detector voltage was 2050 V. Calibration was performed with the All-in-One peptide mix (Ciphergen Biosystems).

Proteinchip software 3.1 with the integrated Biomarker Wizard™ cluster analyses software (Ciphergen Biosystems) [21] was used for data analysis. The baseline was subtracted and profiles were normalized using total ion current before further analyses. Using the cluster analyses tool, peaks (clusters) with signal to noise ratios (S/N) higher than 5 were selected from the eight spectra. This yields the total number of peaks present in one or more of the eight spectra. For each cluster, it is listed in how many spectra a peak is detected with a S/N>5. In this way, the fraction of peaks that is detectable in all 8 spectra with a S/N>5 was calculated as an indicator of the number of high signal to noise peaks detected with a certain chip type/matrix combination. As a measure of spot to spot variation the average coefficient of variation (CV) of peak intensities was calculated for those peaks that were detected in all 8 spectra with a S/N>5.

## Abbreviations

SELDI-TOF-MS, Surface Enhanced Laser Desorption/Ionization-Time-of-flight-Mass Spectrometry; SPA, sinapinic acid ; CHCA, α-Cyano-4-hydroxycinnamic acid; TFA, Trifluoroacetic acid; ACN, Acetonitril; PBS, Phosphate buffered saline; S/N, Signal to Noise ratio; CV, coefficient of variation; CM10, weak cation exchange; WCX, weak cation exchange; Q10, strong anion exchange; IMAC-Cu, copper-coated immobilized metal affinity capture; H50, reverse phase; H4, reversed phase; NP20, normal phase (silica); SEND, Surface Enhanced Neat Desorption.

## Authors' contributions

HR participated in the design of the study and drafted the manuscript. GAL participated in data analyses and helped to draft the manuscript. MS and KL carried out all the SELDI-TOF experiments. RJV participated in the design of the study and helped to draft the manuscript.
